# Suicidal behaviours among in-school adolescents in Mozambique: Cross-sectional evidence of the prevalence and predictors using the Global School-Based Health Survey data

**DOI:** 10.1371/journal.pone.0236448

**Published:** 2020-07-24

**Authors:** Abdul-Aziz Seidu, Hubert Amu, Louis Kobina Dadzie, Abigail Amoah, Bright Opoku Ahinkorah, Edward Kwabena Ameyaw, Henry Yaw Acheampong, Kwaku Kissah-Korsah

**Affiliations:** 1 Department of Population and Health, University of Cape Coast, Cape Coast, Ghana; 2 College of Public Health, Medical and Veterinary Sciences, James Cook University, Townsville, Queensland, Australia; 3 Department of Population and Behavioural Sciences, School of Public Health, University of Health and Allied Sciences, Hohoe, Ghana; 4 The Australian Centre for Public and Population Health Research (ACPPHR), Faculty of Health, University of Technology Sydney, Sydney, Australia; 5 Department of Education and Psychology, Faculty of Educational Foundations, College of Education Studies, University of Cape Coast, Cape Coast, Ghana; University of the Witwatersrand, SOUTH AFRICA

## Abstract

**Introduction:**

Despite interventions by low and middle-income countries toward the achievement of the global Sustainable Development Goal (SDG) on promoting mental health and well-being of their populace by the year 2030, suicidal behaviours continue to be major causes of premature mortality, especially among young people. This study examined the prevalence and predictors of suicidal behaviours among in-school adolescents in Mozambique.

**Materials and methods:**

This was a cross-sectional study of 1918 in-school adolescents using data from the 2015 Global School-based Health Survey (GSHS) of Mozambique. The outcome variables (suicidal ideation, suicidal plan, and suicidal attempt) were measured with single items in the survey. Both bivariate and multivariate analyses were performed using chi-square test of independence and binary logistic regression respectively. Results are presented as Adjusted Odds Ratios for the binary logistic regression analysis. Statistical significance was set at p<0.05.

**Results:**

The prevalence of suicidal behaviours 12 months prior to the survey were 17.7%, 19.6% and 18.5% for suicidal ideation, suicidal plan, and suicidal attempt respectively. Adolescents who experienced anxiety had higher odds of suicidal ideation [AOR = 1.616, 95%CI = 1.148–2.275], suicidal plan [AOR = 1.507, 95%CI = 1.077–2.108], and suicidal attempt [AOR = 1.740, 95%CI = 1.228–2.467]. Adolescents who were physically attacked in school were also more likely to ideate [AOR = 1.463, 95%CI = 1.115–1.921], plan [AOR = 1.328, 95%CI = 1.020–1.728], and attempt [AOR = 1.701, 95%CI = 1.306–2.215] suicide. Having close friends was, however, an important protective factor against suicidal ideation [AOR = 0.694, 95%CI = 0.496–0.971], plan [AOR = 0.625, 95%CI = 0.455–0.860], and attempt [AOR = 0.529, 95%CI = 0.384–0.729]. Peer support also reduced the risk of suicidal ideation [AOR = 0.704, 95%CI = 0.538,0.920] and plan [AOR = 0.743, 95%CI = 0.572,0.966] among the in-school adolescents.

**Conclusion:**

Suicidal behaviours constitute major public health challenges among in-school adolescents in Mozambique. The behaviours are predominant among adolescents who are physically attacked and those who experience anxiety. Conversely, having close friends serves as a protective factor against suicidal behaviours. To ensure that Mozambique meets the SDG target of promoting the mental health of all by the year 2030, the Government of Mozambique and educational authorities should urgently design and implement innovative interventions and strengthen existing ones that seek to address physical attacks and anxiety among in-school adolescents. School administrations should also incorporate programmes that seek to congregate students and offer platforms for social interaction and cohesion.

## Introduction

Despite efforts by countries across the globe to achieve the Sustainable Development Goal (SDG) 3.4 target of promoting the mental health and well-being of their populace by the year 2030 [1], suicidal behaviours remain a major public health concern [2]. Suicidal behaviours entail the spectrum of thoughts that include suicidal ideation, plan, and attempt through fatal completion of suicide which is one of the major causes of premature mortality [3,4]. Globally, about 800,000 people die from suicide every year and 79% of these deaths occur in low- and middle-income countries (LMICs) [5]. Among young people especially adolescents, suicide accounts for an estimated 6% of all deaths [6]. A study conducted among adolescents by Silva et al. [7] reported the prevalence of suicidal ideation, plan, and attempt among adolescents in Brazil as 14%, 9.5%, and 5.9% respectively. In a study conducted among in-school adolescents in LMICs, Uddin et al. [8] found the overall prevalence of suicidal ideation, plan, and attempt as 16.9%, 17.0%, 17.0% respectively.

In sub-Saharan Africa, annual mortality from suicide is estimated at over 34,000 in the general population [9]. Among in-school adolescents, a Ghanaian study by Asante et al. [10] found the prevalence of suicidal ideation, plan, and attempt to be 18.2%, 22.5% and 22.2% respectively. Dunlavy et al. [11] also reported in a Tanzanian study that 7% of in-school adolescents had thought about committing suicide, but 6.3% devised a plan to carry out the suicide. Randall et al. [12] also reported in a Benin study that 23.2% of adolescents had ideated suicide and 28.3% attempted committing suicide. The prevalence of suicidal ideation and attempt among adolescents in Ethiopia were also estimated to be 22.5% and 16.2% respectively [13]. There is, however, a paucity of the empirical literature on the prevalence of suicidal behaviours among adolescents in Mozambique which warranted the conduct of the current study.

Piloted in 1999 with nationwide scale-up in 2007, the Programa Geração Biz (“busy generation”) (PGB) has been a multi-sectoral intervention [14] in Mozambique implemented by the Ministry of Youth and Sports, Ministry of Education, and Ministry of Health to address health needs of adolescents including suicidal behaviours [15]. While it helped to improve healthcare facility visits by adolescents among other achievements [16,17], challenges persist in its operations. For instance, complexity with the multi-sectoral approach, inadequate resources, sparse staffing, and social norms induced resistance militated against the effective implementation of the PGB [14].

Regarding school enrollment, the World Bank [18] indicates that in 2015, primary school enrollment (% net) in Mozambique was 93.93%. Comparatively, while enrollment was 95.38% among males, female primary school enrollment was 92.48%. For secondary education, the total enrollment (% net) was 19.28% in 2015. This was specifically 19.23% for males and 19.32% among females [18].

Suicidal behaviours are influenced by cultural, environmental, genetic, and psychiatric factors [19–21]. According to Kokkevi et al. [22], the major risk factors of suicidal behaviours among adolescents include being a female, exposure to bullying and violence, alcohol and drug use, mental disorders, as well as weak family and peer relationships. Amare et al. [13] also found that school absenteeism, poor social support, and experience of violence are key predictors of suicidal ideation and attempt. Parellada et al. [23] also indicated that suicidal attempts among adolescents are influenced by the physical changes and varied emotional experiences in their environment. Evidence also suggests that parent-child relationships are associated with suicidal behaviours among adolescents [10]. Specifically, some studies have found that parental involvement could serve as protective factors against suicidal behaviours among adolescents. For example, parental factors such as parental bonding (parental knowledge on children’s free time), parental connectedness (understanding of children’s problems), parental supervision (checking children’s homework) have been shown to be protective against adolescents’ suicidal behaviours [10,12,24].

In Mozambique, however, the empirical literature on the predictors of suicidal behaviours among adolescents is limited. The current study, therefore, examined suicidal behaviours and their predictors among in-school adolescents in Mozambique. The findings will help in bridging the literature gap and inform policy decisions regarding the planning and implementation of interventions that will contribute to the mitigation of suicidal behaviours among in-school adolescents.

## Materials and methods

### Description of the survey

This was a cross-sectional study which used data from the 2015 Mozambique Global School-based Health Survey (GSHS) collected using a clustered sample design. It was carried out by the World Health Organization (WHO) in collaboration with the United Nations Children’s Fund (UNICEF), United Nations Educational, Scientific and Cultural Organization (UNESCO), and The Joint United Nations Programme on HIV and AIDS (UNAIDS) with technical assistance from the Centres for Diseases Control and Prevention (CDC) [25]. The GSHS aims to provide data on health and social behaviours among in-school adolescents. The Mozambique GSHS was a school-based survey of students in Class 8–12, which are typically attended by students aged 13–17. A two-stage cluster sample design was used to sample participants who were representative of all students in Class 8–12 in Mozambique. The initial stage of the sampling was characterized by the selection of schools with probability proportional to enrolment size. This was followed by randomly selecting classes and all students in selected classes were eligible to participate in the study. The Mozambique GSHS measured alcohol use, dietary behaviours, drug use, hygiene, mental health, physical activity, protective factors, sexual behaviours, tobacco use, violence and unintentional injury. The students answered the survey questionnaires on a computer scannable answer sheet. The school response rate was 97%, the student response rate was 83%, and the overall response rate was 80%. A total of 1,918 students participated in the survey and were all included in our analysis. Before the commencement of the survey, permission to carry out the study was obtained from the Ministries of Health and Education. Informed consent to participate in the study was obtained from school managers and students. Students anonymously and voluntarily completed the questionnaire. The dataset is freely available for download at https://www.who.int/ncds/surveillance/gshs/mozambiquedataset/en/. We followed the ‘Strengthening the Reporting of Observational Studies in Epidemiology’ (STROBE) statement in conducting this study and writing the manuscript (see S1 Table).

### Study variables

The study considered three outcome variables. These were suicidal ideation, suicidal plan and suicidal attempts [8,9,11,24]. From the dataset, each of these three variables was measured with a single self-reported item or question. The item, “during the past 12 months, did you ever seriously consider attempting suicide?” was used to measure suicidal ideation while suicidal plan was measured with the question, “during the past 12 months, did you make a plan about how you would attempt suicide?” The responses for suicidal ideation and suicidal plan were categorised as “yes” (1) or “no” (0). Suicidal attempt was measured with the question “during the past 12 months, how many times did you actually attempt suicide?” The responses for these questions were “0”, “1”, “2 or 3”, “4 or 5”, and “6 or more times”. The responses were, however, recoded as no attempt (0) and one or more attempts (1) for analysis. Twenty explanatory variables were used to determine their predictive effects on the three outcome variables. These were chosen based on previous studies [10,13,24,26,27]. Detailed descriptions of the variables are presented in S2 Table.

### Data analyses

The 2015 Mozambique GSHS dataset has more than 5% missing values that are missing at random (MAR) pattern. To account for the consistency of the dedicated sample size values of each variable throughout the analyses, we adopted a multiple imputation method to handle the missing values [10, 24]. A full conditional specification method was used after automatic command scanned for the data which were missing at random. A maximum of five imputations were run to allow for > 97% efficiency [24]. Data analyses were performed using STATA version 14.2 software for Mac OS. Due to the nature of the study design, a weighting factor was used in the analysis to reflect the likelihood of sampling each pupil and to reduce bias by compensating for differing patterns of nonresponse. A descriptive analysis was done to describe the general characteristics of the study population, the prevalence of the three outcomes and reported p-values of Pearson’s Chi-Square (bivariate analysis). At the bivariate analysis stage, due to multiple-testing, we introduced a correction method by using the Bonferroni correction method. This was done by dividing the alpha rate (p = 0.05) by the number of analysis performed (20 explanatory variables). Thus, 0.05/20 = 0.0025. Therefore, at the bivariate analysis, statistical significance was declared at p≤0.003. Multicollinearity was checked with the Variance Inflation Factor (Mean VIF = 1.21, Maximum VIF = 1.88, Minimum VIF = 1.02). All the statistically significant variables (p-value p≤0.003) were included in the multivariate analyses. The multivariate analyses in the form of binary logistic regression models were used to determine the strength of association between the explanatory and the outcome variables. The results from the regression analyses were presented as adjusted odds ratios (AOR). The reference categories for all the explanatory variables were informed by previous studies [9,12,19,20] and a priori. The two-sided 95% confidence intervals are reported.

## Results

### Bivariate results

Table 1 presents results on the relationship between the explanatory variables and suicidal behaviours among adolescents in Mozambique. The prevalence of suicidal behaviours were 17.7%, 19.6% and 18.5% for suicidal ideation, suicidal plan and suicidal attempt, respectively. The chi-square analyses showed that the number of close friends, anxiety, experiencing hunger, being bullied, engaging in a fight, being attacked, and sustaining an injury were significantly associated with suicidal ideation, plan and attempt. Age and truancy and were only statistically associated with suicidal ideation. Loneliness, tobacco use, and parental connectedness only showed statistically significant association with suicidal plan and attempt. Age was only associated with suicidal ideation, smoke was only associated with suicidal attempt, while parental bonding was only associated with the suicidal plan.

**Table 1:** Bivariate relationship between explanatory variables and suicidal behaviours among adolescents in Mozambique.

Variables	Suicidal Ideation			Suicidal plan			Suicidal attempt		
	No	Yes	P-value	No	Yes	P-value	No	Yes	P-value
**Prevalence**	82.3	17.7		80.4	19.6		81.5	18.5	
**Age**			0.048			0.134			0.284
11–14	85.83	14.17		83.16	16.84		83.42	16.58	
15+	81.48	18.52		79.73	20.27		81.02	18.98	
**Sex**			0.162			0.139			0.348
Female	81.06	18.94		79.00	21.00		80.63	19.37	
Male	83.50	16.50		81.69	18.31		82.29	17.71	
**Grade**			0.328			0.750			0.503
8 to 10	82.83	17.17		80.23	19.77		81.14	18.86	
11+	80.89	19.11		80.89	19.11		82.49	17.51	
**Close friends**			0.001			<0.001			<0.001
0 friends	74.90	25.10		71.26	28.74		69.23	30.77	
1 or more friends	83.42	16.58		81.75	18.25		83.30	16.70	
**Anxiety**			<0.001			<0.001			<0.001
No	83.50	16.50		81.51	18.49		83.09	16.91	
Yes	72.73	27.27		71.29	28.71		68.42	31.58	
**Loneliness**			0.037			0.068			0.022
No	82.95	17.05		80.97	19.03		82.19	17.81	
Yes	77.07	22.93		75.61	24.39		75.61	24.39	
**Tobacco**			0.001			0.158			<0.001
No	82.93	17.07		80.65	19.35		82.55	17.45	
Yes	67.12	32.88		73.97	26.03		54.79	45.21	
**Alcohol**			0.571	80.19	19.81	0.545			0.981
No	82.51	17.49		81.86	18.14		81.50	18.50	
Yes	81.01	18.99					81.43	18.57	
**Smoke**			0.110			0.489			0.003
No	82.54	17.46		80.30	19.70		81.90	18.10	
Yes	73.33	26.67		84.44	15.56		64.44	35.56	
**Experienced hunger**			0.001			0.029			<0.001
No	85.12	14.88		82.22	17.78		84.35	15.65	
Yes	79.05	20.95		78.26	21.74		78.14	21.86	
**Bullied**			0.004			0.002			<0.001
No	84.41	15.59		82.66	17.34		85.90	14.10	
Yes	79.25	20.75		77.06	22.94		75.00	25.00	
**Fight**			<0.001			<0.001			<0.001
No	84.55	15.45		82.43	17.57		84.85	15.15	
Yes	76.81	23.19		75.36	24.64		73.19	26.81	
**Attack**			<0.001			<0.001			<0.001
No	85.44	14.56		83.14	16.86		86.05	13.95	
Yes	75.69	24.31		74.55	25.45		71.78	28.22	
**Injury**			<0.001			<0.001			<0.001
No	85.60	14.40		85.12	14.88		87.14	12.86	
Yes	78.42	21.58		74.77	25.23		74.77	25.23	
**Truancy**			0.006			0.249			0.337
No	83.74	16.26		81.01	18.99		81.99	18.01	
Yes	78.21	21.79		78.62	21.38		80.04	19.96	
**Sedentary**			0.463			0.665			0.838
No	81.86	18.14		80.11	19.89		81.62	18.38	
Yes	83.21	16.79		80.94	19.06		81.24	18.76	
**Peer support**			0.001			0.002			0.057
No	76.89	23.11		75.33	24.67		78.44	21.56	
Yes	83.99	16.01		81.95	18.05		82.43	17.57	
**Parental supervision (homework)**			0.440			0.509			0.282
No	81.50	18.50		79.66	20.34		80.31	19.69	
Yes	82.87	17.13		80.88	19.12		82.27	17.73	
**Parental Connectedness (understanding)**			0.341			0.038			0.026
No	81.60	18.40		78.75	21.25		79.76	20.24	
Yes	83.27	16.73		82.55	17.45		83.75	16.25	
**Parental Bonding (free time)**			0.258			0.032			0.260
No	81.19	18.81		78.16	21.84		80.34	19.66	
Yes	83.18	16.82		82.08	17.92		82.36	17.64	

Source: 2015 Mozambique GSHS

### Multivariate results

Table 2 presents results from the multivariate logistic regression analyses on the predictors of suicidal ideation, plan and attempt. Concerning suicidal ideation, adolescents who experienced anxiety [AOR = 1.616,95%CI = 1.148,2.275] and hunger [AOR = 1.321, 95%CI = 1.029,1.697], those who were physically attacked [AOR = 1.463, 95%CI = 1.115,1.921], and sustained injuries [AOR = 1.319, 95%CI = 1.024,1.700] had higher odds of suicidal ideation. Adolescents who had close friends [AOR = 0.694, 95%CI = 0.496,0.971] and whose peers were supportive [AOR = 0.704, 95%CI = 0.538,0.920] had lower odds of suicidal ideation. With suicidal plan, those who were anxious [AOR = 1.507, 95%CI = 1.077,2.108], attacked [AOR = 1.328, 95%CI = 1.020,1.728] and who sustained injuries [AOR = 1.643, 95%CI = 1.285,2.101] had higher odds of planning suicide. Adolescents who had close friends [AOR = 0.625, 95%CI = 0.455,0.860] and supportive friends [AOR = 0.743, 95%CI = 0.572,0.966] were, however, less likely to plan suicide. For suicidal attempt, adolescents who were anxious [AOR = 1.740, 95%CI = 1.228,2.467]], those who used tobacco [AOR = 2.162, 95%CI = 1.170,3.994], those who were physically attacked [AOR = 1.701, 95%CI = 1.306,2.215], bullied [AOR = 1.461[1.139,1.873], and sustained injuries [AOR = 1.659, 95%CI = 1.288,2.137] were more likely to attempt suicide. On the contrary, adolescents who had close friends [AOR = 0.529, 95%CI = 0.384,0.729] had lower odds of attempting suicide.

**Table 2:** Multivariate logistic regression analyses on the predictors of suicidal behaviours among adolescents in Mozambique.

Variables	Suicidal ideation AOR (95%CI)	p-value	Suicidal plan AOR (95%CI)	p-value	Suicidal attempt AOR (95%CI)	p-value
Close friends *(Ref = No)*	0.694[0.496–0.971]	0.033	0.625[0.455–0.860]	0.004	0.529[0.384–0.729]	<0.001
Anxiety *(Ref = No)*	1.616[1.148–2.275]	0.006	1.507[1.077–2.108]	0.017	1.740[1.228–2.467]	0.002
Tobacco use *(Ref = No)*	1.444[0.850–2.452]	0.174	–	–	2.162[1.170–3.994]	0.014
Experienced Hunger *(Ref = No)*	1.321[1.029–1.697]	0.029	1.112[0.875–1.414]	0.384	1.193[0.928–1.535]	0.168
Physical fight *(Ref = No)*	1.201[0.913–1.580]	0.191	1.125[0.861–1.469]	0.389	1.266[0.970–1.653]	0.082
Attacked *(Ref = No)*	1.463[1.115–1.921]	0.006	1.328[1.020–1.728]	0.035	1.701[1.306–2.215]	<0.001
Injured *(Ref = No)*	1.319 [1.024–1.700]	0.032	1.643[1.285–2.101]	<0.001	1.659[1.288–2.137]	<0.001
Peer support *(Ref = No)*	0.704[0.538–0.920]	0.010	0.743 [0.572–0.966]	0.026	–	–
Bullied *(Ref = No)*	–	–	1.121[0.882–1.426]	0.351	1.461[1.139–1.873]	0.003
Smoke *(Ref = No)*	–	–	–	–	0.881[0.365–2.127]	0.779
N	1918		1918		1918	
Pseudo R^2^	0.038		0.036		0.078	

AOR = adjusted odds ratio, CI = Confidence Interval

Source: 2015 Mozambique GSHS

## Discussion

This was a cross-sectional study of in-school adolescents based on the 2015 GSHS of Mozambique. The prevalence of suicidal behaviours were 17.7%, 19.6% and 18.5% for suicidal ideation, suicidal plan and suicidal attempt respectively. These findings are consistent with those reported in Ghana [10], Benin [12] and Ethiopia [13]. However, the prevalence in the current study are relatively higher than what was reported in Nepal [28] and Tanzania [11]. The possible reasons accounting for the variations in study findings could be the differences in the measurement of suicidal behaviours as well as differences in time and study settings. For example, Asante et al. [10] in Ghana and Randal et al. [12] in Benin reported 12 months-prevalence. However, Amare et al. [13] reported lifetime prevalence. Also, Cheng et al. [29] indicated that there are complexities surround the factors that predispose an individual to suicidal behaviours in various environments.

We found that adolescents who went hungry in school were at a higher risk of ideating suicide. This points to the overarching role of poverty in adversely affecting the general school performance of adolescents which in the case of the present study is related to their mental health [30,31]. The finding confirms previous studies which have argued that poor adolescents who go hungry in school are more likely to report suicidal behaviours than the rich ones [32,33]. The fact that hungry adolescents had higher odds of ideating suicide could be due to the psychological strain hunger exerts on their mental health [34].

Being physically attacked and sustaining injury were all risk factors for suicidal ideation, plan, and attempt among in-school adolescents in the present study. This finding corroborates other studies which posited that suicidal behaviours among adolescents are related to having a history of physical abuse [10,35,36]. The adolescents might have ideated, planned, and attempted suicide after being physically abused and sustaining injuries due to trauma, cognitive distortions, and humiliation they might have experienced which they may have to live with for the rest of their lives [37]. This reiterates the need for Mozambican authorities to implement innovative interventions and strengthen existing ones that seek to support victims of physical attack to ensure achievement of SDG goal 3.4 by the year 2030.

Our study revealed that bullying is a significant risk factor for suicidal attempt among in-school adolescents. This justifies arguments that bullying has become lethal and pervasive in schools, deprives students of their chances to learn, and exacts mental scars that last throughout their lifetime [38–40]. Our findings regarding bullying are congruent to Alavi et al.’s [41] postulations that adolescents with suicidal attempt are more likely to report ever being bullied. Van Geel et al. [42] also argued that all forms of bullying are importantly linked with increases in suicidal attempt among adolescents. Our finding regarding suicidal plan and attempt among bullied adolescents could be due to the potential psychological problems including stress, anxiety and depression that they might have experienced. This is because, several previous studies have argued that adolescents who are bullied publicly in front of their peers develop a sense of shame and dishonour from the victimization and further exposes them to episodes of stress, anxiety, and depression which in turn could induce them to attempt suicide [43–46]. Our finding of anxiety as an important predictor of suicidal behaviours is indicative of this assertion. There is, therefore, the need for authorities and other stakeholders to institute or strengthen existing measures to tackle bullying in schools as well as rehabilitative measures to support psychologically distressed adolescents, to help achieve the global agenda of SDG 3.4 which entreats all governments across LMICs to promote mental health and well-being by the year 2030 [1].

We realised that having close friends served as a protective factor against suicidal ideation, plan and attempt among adolescents. In-school adolescents who had support from their peers were also protected from experiencing suicidal plan and attempt. The findings regarding close friends and peer support highlight the role of social support in mitigating the adverse effects of mental health challenges on the psychosocial development of adolescents [47–49]. Our finding could be because adolescents mostly turn to their close friends as their source of emotional, financial, advice and other possible supports during a time of crisis or need [50,51]. This is probably because they find it easy and safe to confine in their close friends regarding their challenges. These challenges include poor academic performance and romantic relationship problems which have negative mental health implications including suicidal behaviours [52–55].

### Strengths and limitations

A key strength of this study is that it is the first empirical effort to comprehensively understand suicidal behaviours among in-school adolescents in Mozambique using the Global School-Based Health Survey data. Recommendations made are thus useful for policy-makers regarding the interventions needed to reduce such behaviours among adolescents especially in school environments. The use of multivariate logisitic regression also ensured that we were able to measure the extent of association that the explanatory variables had on the outcome variables while controlling for several confounders. Additionally, since the study has used the globally standardized methodology of the Global School-Based Health Survey, study findings are also comparable to other countries adopting the same or similar methodology [28].

Despite the important findings and the rigour involved in our analyses, the study results should be interpreted with these limitations in mind. First, our study shares all the shortfalls associated with cross-sectional study designs more especially regarding the establishment of causality. Secondly, there is the possibility of recall and social desirability biases from the adolescents [10,28]. Thirdly, the fact that our study focused on only in-school adolescents means that the results are not generalizable to all adolescents in the country [24]. Moreover, since the study used secondary data, GSHS, information on other variables such as socioeconomic status, religious affiliation, social participation and psychological co-morbidities that could be important in characterizing suicidal behaviours could not be assessed [28]. Additionally, family history of suicidal behaviours and the number of guardians/parents were not available in the dataset [24]. However, evidence suggests that traces and experiences of suicidal behaviours among other family members, family discord, death of a parent as well as divorce increase the risk of suicidal behaviours among adolescents [56]. Adding these variables could have further strengthened our study. Furthermore, all the outcome variables were measured with single items. In terms of missing values of the outcome variables, 92 (4.80%), 94(4.9%), and 57 (2.9%) responses for suicidal ideaion, plan and attempt were respectively missing and were imputed using multiple imputation method.

## Conclusion

Suicidal behaviours constitute major public health challenge among in-school adolescents in Mozambique. Adolescents most at risk of these behaviours are those who are bullied, physically attacked, those who sustain injuries and adolescents who go hungry in school. Having close friends and getting support from peers, however, protect in-school adolescents against the risk of suicidal behaviours. To ensure that Mozambique meets the SDG 3 target of promoting the mental health of all by the year 2030, the Government and educational authorities of the country should urgently design and implement innovative interventions as well as strengthen existing ones that seek to address bullying, physical attack, and injury among adolescents. School administrations should also incorporate programmes that seek to congregate students and offer platforms for social interactions and cohesion.

## Supporting information

S1 Table STROBE statement—Checklist of items that should be included in reports of *cross-sectional studies*. (DOC)

S2 Table Study variables. (DOCX)

S3 Table Multicollinearity test. (DOCX)

## Abbreviations

AOR: Adjusted Odds Ratios
CI: Confidence Interval
GSHS: Global School-based Health Survey
LMICs: low- and middle-income countries
SDGs: Sustainable Development Goals
WHO: World Health Organisation
