# Supplementary material for: Suicidal behaviours among in-school adolescents in Mozambique: Cross-sectional evidence of the prevalence and predictors using the Global School-Based Health Survey data
Source: PLoS One. 2020 Jul 24;15(7):e0236448. doi: 10.1371/journal.pone.0236448 (PMC7380623; doi:10.1371/journal.pone.0236448)
Supplement: S2 Table — (DOCX) [file pone.0236448.s002.docx]

**Table S2. ﻿ Study variables**

| **Variables** | **Question** | **Response options and recoding** |
| --- | --- | --- |
| **Outcome variables** | | |
| Suicidal ideation | During the past 12 months, did you ever seriously consider attempting suicide? | 1 = Yes 2 = No  **Coded as 1 = Yes and 0=No)** |
| ﻿Suicide plan | During the past 12 months, did you make a plan about how you would attempt suicide? | 1 = Yes 2 = No  **(coded as 1 = Yes and 0=No)** |
| Suicidal Attempt | During the past 12 months, how many times did you actually attempt suicide? | “0”, “1”, “2 or 3”, “4 or 5”, and “6 or more times”.  **(coded as 0=no attempt and 1=one or more attempts** |
|  | | |
| **Explanatory variables** | | |
| Age | Custom age | 1=12, 2=13, 3=14, 4=15, 5=16, 6=17, 7=18 years  **(coded as 0=12-17, 18+=1)** |
| Sex | Sex | 1=male, 2=female |
| Grade | In what grade are you? | 1=SHS1, 2=SHS2, 3=SHS3, 4=SHS4 |
| Tobacco use | ﻿During the past 30 days, on how many days did you use any other form of tobacco, such as chewing  tobacco leaves? | 1 = 0 days; to 7 = All 30 days  **(coded as 1 = 0; and 2–7 = 1)** |
| Alcohol use | ﻿During the past 30 days, on how many days did you have at least one drink containing alcohol? | ﻿1 = 0 days; to 7 = All 30 days  **(coded as 1 = 0; and 2–7 = 1)** |
| Smoking | ﻿During the past 30 days, how many days did you smoke cigarette? | 1 = 0 days; to 7 = All 30 days  **(coded as 1 = 0; and 2–7 = 1)** |
| Loneliness | ﻿During the past 12 months, how often have you felt lonely? | ﻿1 = never, 2 = rarely, 3 = sometimes, 4 = most of the time to 5 = always  (**coded as 1**-3 **= 0 and** 4**- 5 = 1)** |
| Anxiety | ﻿During the past 12 months, how often have you been so worried about something that you could not sleep at night? | ﻿1 = never to 5 = always  **(coded 1 - 3 = 0 and 4 – 5 = 1)** |
| Truancy | ﻿During the past 30 days, on how many days did you miss classes or school without permission? | 1=0 days,2= 1or 2 days, 3=3 to 5 days, 4=6 to 9 days, 5= 10 or more  (**coded 1=0 and 2-5=1)** |
| Hunger (proxy of socioeconomic status) | Went hungry past 30 days | 1=never, 2=Rarely, 3=sometimes, 4=most of the times, 5=always  **(coded 1-3=0, 4-5=1)** |
| Bullied | ﻿During the past 30 days, how were you bullied most often? | ﻿1 = 0 times; to 8 = 12 or more times  **(coded 1 = 0; and 2–7 = 1)** |
| Fight | ﻿During the past 12 months, how many times were you in a physical fight? | ﻿﻿1 = 0 times; to 8 = 12 or more times  (**coded 1 = 0; and 2–8 = 1)** |
| Injury | ﻿During the past 12 months, how many times were  you seriously injured? | ﻿1 = 0 times; to 8 = 12 or more time  **(coded as 1 = 0; and 2–8 = 1)** |
| Attacked | ﻿During the past 12 months, how many times were you physically attacked? | 1=0 days,2= 1or 2 days, 3=3 to 5 days, 4=6 to 9 days, 5= 10 or more  (**coded 1=0 and 2-5=1)** |
| ﻿﻿Sedentary behaviour | ﻿How much time do you spend during a typical or usual day sitting and watching television, playing computer games, talking with friends, or doing other sitting activities?” | ﻿﻿1 = less than 1 h per day, 2=1-2 hours, 3 = 3–4 h per day, 4=5-6 h per day, 5=7-8 h per day, 6 = 8 or more hours a day  **Coded as:(1-2=No, 2-8=Yes)** |
| Close friends | ﻿How many close friends do you have? | 1=0 to 4=3 or more  **(coded as 1=0, 1-2=1, 3 or more=2)** |
| Helpful  (Peer support) | ﻿During the past 30 days, how often were most of the students in your school kind and helpful? | 1=never, 2=Rarely, 3=sometimes, 4=most of the times, 5=always  **(coded 1-3 = 1; and 4–5 = 1)** |
| Parents check homework  (parental supervision) | ﻿During the past 30 days, how often did your parents or  guardians check to see if your homework was done? | 1=never, 2=Rarely, 3=sometimes, 4=most of the times, 5=always  **(coded 1-3 = 0; and 4–5 = 1)** |
| Understand problems  ﻿(Parental  Connectedness | ﻿During the past 30 days, how often did your parents or guardians understand your problems and worries? | ﻿1=never, 2=Rarely, 3=sometimes, 4=most of the times, 5=always  **(coded 1-3 = 0; and 4–5 = 1)** |
| Know what adolescent do free time (﻿Parental or guardian  Bonding) | ﻿During the past 30 days, how often did your parents or guardians really know what you were doing with your free time? | ﻿1=never, 2=Rarely, 3=sometimes, 4=most of the times, 5=always  **(coded 1-3= 0; and 4–5 = 1)** |
