# Supplementary material for: Suicidal behaviours among in-school adolescents in Mozambique: Cross-sectional evidence of the prevalence and predictors using the Global School-Based Health Survey data
Source: PLoS One. 2020 Jul 24;15(7):e0236448. doi: 10.1371/journal.pone.0236448 (PMC7380623; doi:10.1371/journal.pone.0236448)
Supplement: S3 Table — (DOCX) [file pone.0236448.s003.docx]

**Table S3. Multicollinearity Test**

| Variable | VIF | SQRT  VIF | Tolerance | R-Squared |
| --- | --- | --- | --- | --- |
| Age | 1.10 | 1.05 | 0.9114 | 0.0886 |
| Sex | 1.04 | 1.02 | 0.9637 | 0.0363 |
| Grade | 1.12 | 1.06 | 0.8900 | 0.1100 |
| Close_friends | 1.08 | 1.04 | 0.9294 | 0.0706 |
| Anxiety | 1.09 | 1.05 | 0.9151 | 0.0849 |
| Loneliness | 1.09 | 1.04 | 0.9169 | 0.0831 |
| Tobacco | 1.88 | 1.37 | 0.5317 | 0.4683 |
| Alcohol | 1.08 | 1.04 | 0.9267 | 0.0733 |
| Smoke | 1.85 | 1.36 | 0.5400 | 0.4600 |
| Hungry | 1.11 | 1.05 | 0.9041 | 0.0959 |
| Bullied | 1.15 | 1.07 | 0.8689 | 0.1311 |
| Fight | 1.26 | 1.12 | 0.7956 | 0.2044 |
| Attack | 1.27 | 1.13 | 0.7899 | 0.2101 |
| Injury | 1.19 | 1.09 | 0.8390 | 0.1610 |
| Truancy | 1.05 | 1.02 | 0.9558 | 0.0442 |
| Sedentary | 1.02 | 1.01 | 0.9814 | 0.0186 |
| Helpful | 1.06 | 1.03 | 0.9430 | 0.0570 |
| Homework | 1.22 | 1.11 | 0.8188 | 0.1812 |
| Understand_problem | 1.21 | 1.10 | 0.8240 | 0.1760 |
| Freetime | 1.30 | 1.14 | 0.7694 | 0.2306 |
| Mean VIF | 1.21 |  |  |  |

The general rule of thumb is that VIFs exceeding 4 warrant further investigation, while VIFs exceeding 10 are signs of serious multicollinearity requiring correction
